# Supplementary material for: GloBIAS: strengthening the foundations of BioImage Analysis
Source: ArXiv. 2025 Aug 25:arXiv:2507.06407v2. Originally published 2025 Jul 8. Preprint. [Version 2] (PMC12265592)
Supplement: Supplement 1 [file NIHPP2507.06407v2-supplement-1.pdf]

Supplementary

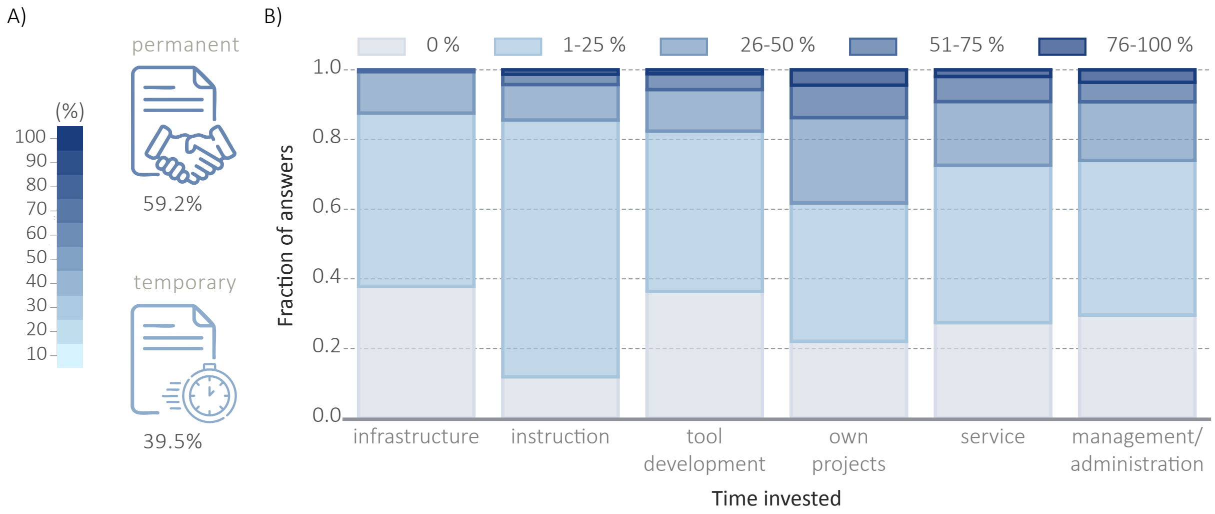

Supplementary Figure 1: Description of respondents current work. A) Percentage of respondents with a permanent or temporary position. **B)** Stacked bar plot showing the percentage of time invested in different tasks as described by respondents.
